# Nasosinusal chondrosarcoma with orbito-cerebral extension

**DOI:** 10.1093/jscr/rjac286

**Published:** 2022-06-22

**Authors:** Khalid Bouhafs, Azeddine Lachkar, Tayeb Bouamama, Achraf Miry, Drissia Benfadil, Mohammed Rachid Ghailan

**Affiliations:** Otorhinolaryngology and Head and Neck Surgery Department, Mohammed VI University Hospital of Oujda, Oujda, Morocco; Otorhinolaryngology and Head and Neck Surgery Department, Mohammed VI University Hospital of Oujda, Oujda, Morocco; Radiology Department, Mohammed VI University Hospital Center of Oujda, Oujda, Morocco; Pathological Anatomy Department, Mohammed VI University Hospital Center of Oujda, Oujda, Morocco; Otorhinolaryngology and Head and Neck Surgery Department, Mohammed VI University Hospital of Oujda, Oujda, Morocco; Otorhinolaryngology and Head and Neck Surgery Department, Mohammed VI University Hospital of Oujda, Oujda, Morocco

## Abstract

Chondrosarcoma is a highly aggressive malignant tumor originating from cartilaginous and mesenchymal tissues. The aim of this report is to describe a rare case of nasosinusal chondrosarcoma with orbito-cerebral extension. Our patient was a 55-year-old with a right cheek swelling evolving over a year, with unilateral right nasal obstruction gradually becoming bilateral associated with hyposmia, bilateral exophthalmos, reduced bilateral deep visual acuity and permanent headaches. The clinical examination found a tumor obstructing the two nasal cavities. Imaging showed a lobulated heterogeneous tissue process occupying the paranasal sinuses, with calcifications and enhancement at its periphery, extending to the orbito-cerebral area. The histopathological analysis was in favor of chondrosarcoma. The patient was first treated with an incomplete surgical resection by an endonasal route due to the extension to the orbit and the brain and received adjuvant radiotherapy. Surgical excision is a prognostic factor in this type of sarcomas and reduces recurrence rates.

## INTRODUCTION

Chondrosarcoma is a primary malignant bone tumor that develops from the cartilage without producing bone tissue [1]. Representing 10–20% of malignant bone tumors [1], it only affects the cervico-facial region in 7% of cases [2]. The nasosinusal location is even rarer. To the best of our knowledge, the published literature reported only 20 cases [3]. In this paper, we report the first case in the Moroccan Oriental region.

## CASE PRESENTATION

**Figure 1:**
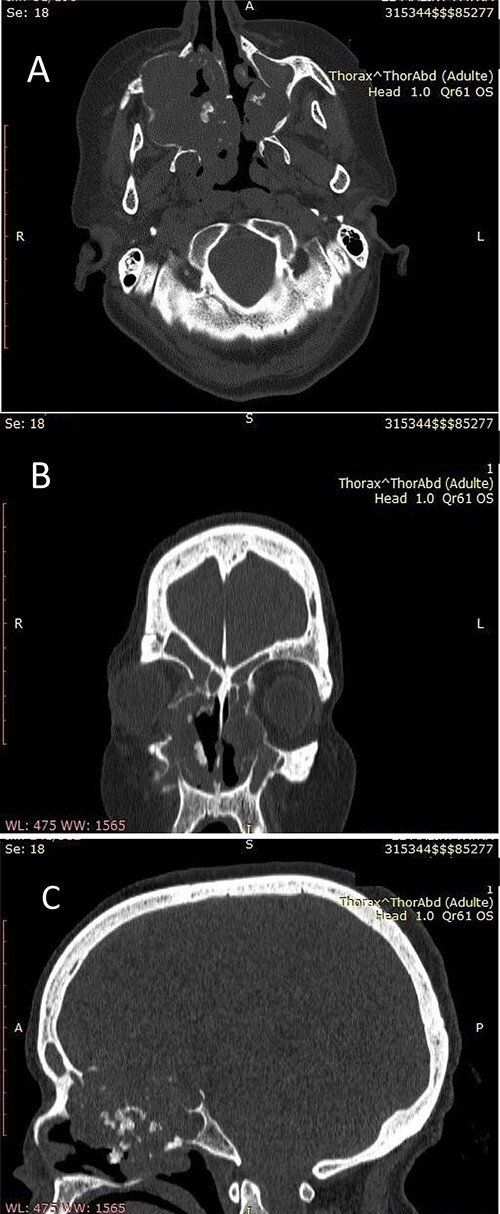
(**A**) Nasosinusal CT in axial section showing an isodense tissue process, located in the right maxillary sinus and the ipsilateral nasal cavity, extended to the right anterolateral wall of the cavum and to the contralateral nasal cavity after lysis of the nasal septum, with calcifications. (**B**) Nasosinusal CT in coronal section showing the extension of the tumor process to ethmoidal cells and to the right orbit after lysis of the papery lamina. (**C**) Nasosinusal CT in sagittal section showing the extension of the tumor process to the ethmoid–sphenoid complex and to the frontal sinuses.

**Figure 2:**
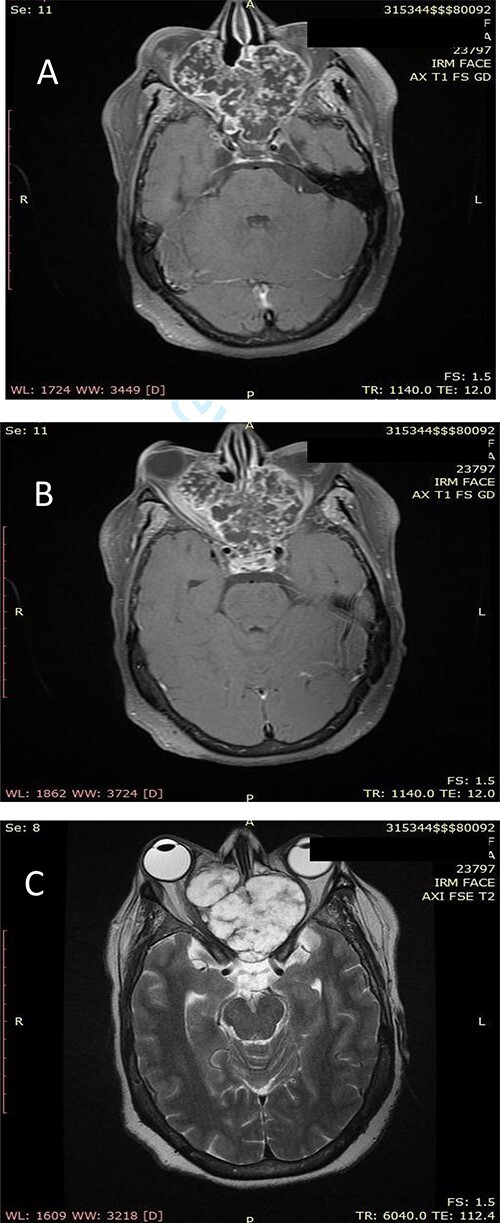
(**A**) Axial facial MRI in T1 sequence after Gadolinium injection showing a lobulated tissue process, occupying the nasal cavities, enhancing heterogeneous. (**B**) Axial facial MRI in T1 sequence after Gadolinium injection showing bilateral exophthalmos and a mass effect on the optic nerves. (**C**) Axial facial MRI in T2 sequence showing endocranial extension opposite the frontal lobes.

**Figure 3:**
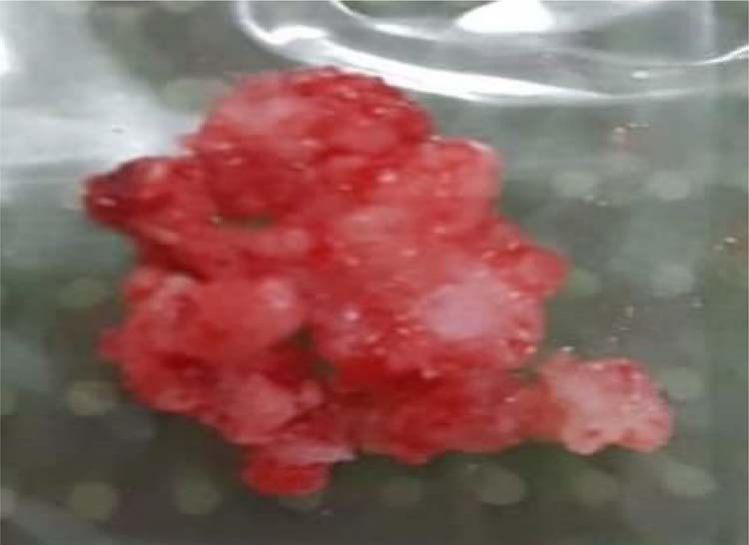
Surgical specimen with cartilage tissue.

**Figure 4:**
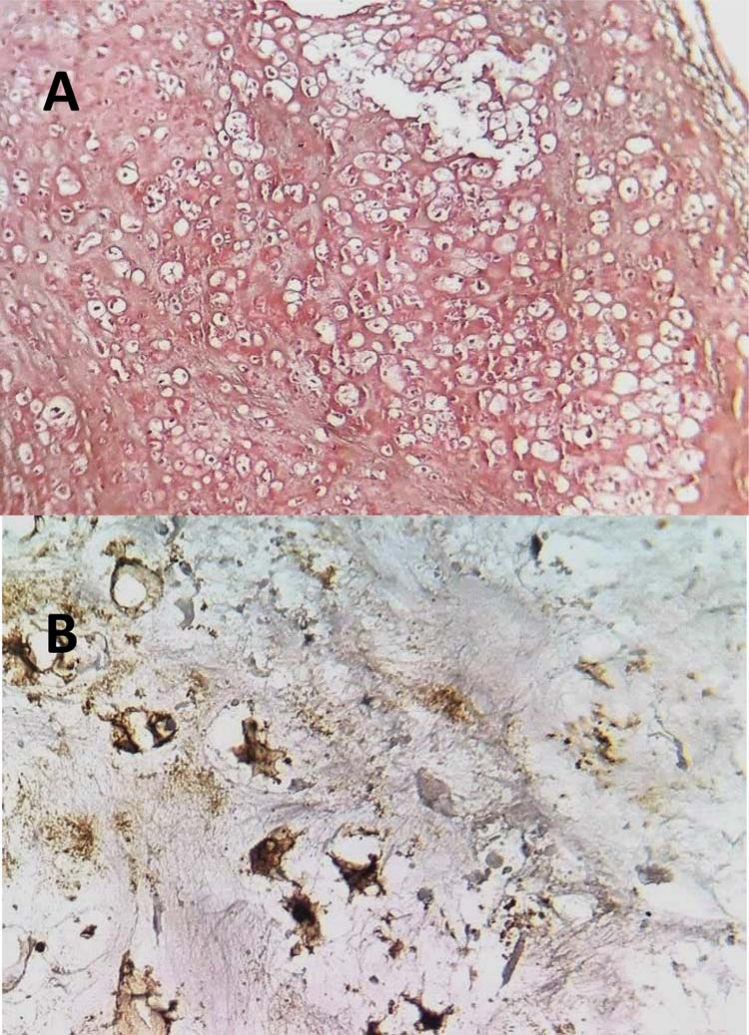
(**A**) The histopathological analysis showing moderately cellular chondrocyte proliferation on a cartilaginous background. Few mitotic figures have been observed. HE; ×100. (**B**) neoplastic cells express the S100 protein.

Our patient was a 55-year-old woman, without significant pathological history, presenting a right cheek swelling evolving over 1 year, with unilateral right nasal obstruction gradually becoming bilateral which was associated with a hyposmia, a bilateral exophthalmos, decrease bilateral deep visual acuity and intense permanent frontal headaches that were resistant to the usual analgesics. The clinical examination found a straight cheek and fixed painful mass of firm consistency, with irregular contours. The nasal flow was abolished on the right side and reduced on the left. Nasal endoscopy revealed a budding tumor process obstructing the two nasal cavities, without bleeding and it was masked by purulent secretions. Craniofacial computed tomography (CT) scan showed a large tumor process in the center of the nasosinusal region containing peripheral and central popcorn calcifications, with irregular contours, weakly enhancing after injection of contrast product and measuring ~65 × 62 mm (Fig. 1A–C; see Supplementary Material 1 for a detailed description). Magnetic resonance imaging (MRI) showed a lobulated, heterogeneous tissue process, hyperintense in T2, hypointense in T1. This tumor had a heterogeneous enhancement after injection of Gadolinium and measured 61 × 60 mm. It was also centering on the nasal cavities and on the ethmoid cells that caused lysis of the nasal turbinates and invaded the riddled blades of the ethmoid and with intra-orbital extension bilaterally (Fig. 2A–C; see Supplementary Material 1 for a detailed description). The histopathological analysis of the biopsy specimens found chondrosarcoma. An additional imaging based on thoraco-abdominal-pelvic CT scan did not show other locations. The patient underwent an endoscopic endonasal surgery that was incomplete due to the extension to the orbit and the brain (Fig. 3). The histopathologial analysis of the resected specimens confirmed the diagnosis of grade 2 chondrosarcoma (Fig. 4A and B). Our patient received adjuvant radiotherapy using a total dose of 50 Gy. After 6 months, post-therapeutic imaging based on CT and MRI showed a significant and clear decrease of the tumor volume with persistent residual tumor at the base of the skull given the incomplete excision. With a follow-up of 9 months, there was no recurrence.

## DISCUSSION

Chondrosarcoma is a heterogeneous group of malignant bone tumors of cartilaginous and mesenchymal origins [2, 4]. The symptoms of chondrosarcomas are nonspecific and vary depending on the site and the invasion of the tumor [5, 6]. Imaging is based on the combination of CT and MRI that show several characteristics in favor of chondrosarcoma including lobulated tumor, with irregular contours, with a destructive behavior, of a density lower than that of the bone and also the presence of calcifications with an enhancement. The bilateral invasion of the oculomotor muscles and the effects of the tumor on the optic nerves explains the bilateral external ophthalmoplegia and blindness reported in our patient. Likewise, the invasion of the frontal sinuses and the endocranial extension in front of the frontal lobes are responsible of the headaches observed in our patient.

The treatment of chondrosarcomas is essentially based on surgical removal of sarcomatous tissues in combination with adjuvant radiotherapy despite the immature evidence supporting its effect on chondrosarcomas. The external route in which a complete tumor resection can be performed but with poor esthetic outcomes and quality of life due to its extension to the adjacent tissues [7]. Furthermore, the endoscopic endonasal route that has become the standard route of choice for certain locations such as the sphenoid because of its advantages especially reduced morbidity and iatrogenicity. Our patient underwent endoscopic endonasal surgery and the resection was incomplete due to the orbit and the endocranial extension and received an adjuvant radiation. The prognosis of chondrosarcomas is poor and depends on several factors such as patients’ age of onset, the primary tumor site, the histopathological grading, the myxoid or mesenchymal variant and the quality of the surgical excision that remains the most important parameter affecting overall survival. The 5-year survival is 44–87% [8–14]. Recurrence is estimated at 40–60% and associated with the prognostic evolution.

## CONCLUSION

Nasosinusal chondrosarcoma is very rare and aggressive. Imaging is of crucial importance in the management of these rare types of sarcomas. The quality of surgery is a determinant factor that affects patients’ outcomes.

## DISCLOSURE

All the authors contributed to the conduct of this work. All authors also declare that they have read and approved the final version of the manuscript.

## CONFLICT OF INTEREST STATEMENT

None declared.

## FUNDING

None.

## DATA AVAILABILITY

The patient’s data are available upon reasonable request from the corresponding author.

## Supplementary Material

Supplementary_material_1_rjac286
