# Supplementary material for: Nasosinusal chondrosarcoma with orbito-cerebral extension
Source: J Surg Case Rep. 2022 Jun 22;2022(6):rjac286. doi: 10.1093/jscr/rjac286 (PMC9216040; doi:10.1093/jscr/rjac286)
Supplement: Supplementary_material_1_rjac286 [file supplementary_material_1_rjac286.docx]

**Supplementary material 1 for:** **Nasosinusal chondrosarcoma with orbito-cerebral extension**

**-Details of clinical examination:**

The ophthalmologic examination showed a negative light perception of the 2 eyes, a bilateral painless non-axile non pulsatile irreducible exophthalmos, a bilateral areflexic mydriasis, external ophthalmoplegia and compressive optic neuropathy. A neurologic examination found an involvement of the first four cranial pairs. The cervical lymph node areas were free.

**-Details of CT scan:**

The tumor was extending over 52mm, laterally invading the internal rectus muscle of the eye bilaterally and was more marked on the right, and with a compressive contact with the optic nerves. Furthermore, it invaded the nasal septum, the vomer, the upper jaw, and the base of the right orbit. This tumor was in contact with the lower rectus muscle and presented an extension to the sella turcica and invaded the ethmoidal labyrinth and the frontal sinuses to spread intracranially (**Figure 1A**, **Figure 1B**, and **Figure 1C**).

**-Details of MRI findings:**

This imaging also showed that this tumor was covering the internal rectus muscles and has a contact with the optic nerves that caused a grade III proptosis on the right and grade II on the left. Below and laterally, it invaded the maxillary sinuses, the alveolar bone and the hard palate on the right side. It also invaded the frontal and the sphenoid sinuses with a lytic impact on the crista galli process with an endocranial extension. Finally, it infiltrated the right anterolateral wall of the cavum (**Figure 2A, Figure 2B, Figure 2C**).
